# Correction of arterial input-function saturation for extraction fraction estimation in retinal fluorescein videoangiography

**DOI:** 10.1088/1361-6560/aeb793

**Published:** 2026-10-30

**Authors:** Sarah R Vavrek, Elif Kayaalp Nalbant, Nicholas Konopek, Nicole L Decker, Amani A Fawzi, William F Mieler, Jennifer J Kang-Mieler, Kenneth M Tichauer

**Affiliations:** 1 Biomedical Engineering, Illinois Institute of Technology, Chicago, IL 60616, United States of America; 2 Department of Ophthalmology, Feinberg School of Medicine, Northwestern University, Chicago, IL 60611, United States of America; 3 Department of Ophthalmology and Visual Sciences, Illinois Eye and Ear Infirmary, University of Illinois at Chicago, Chicago, IL 60607, United States of America; 4 Department of Biomedical Engineering, Stevens Institute of Technology, Hoboken, NJ 07030, United States of America; 5 Department of Medical Biophysics, Western University, London, ON N6A 5C1, Canada; 6 Lawson Research Institute, St. Joseph’s Health Care, London, ON N6A 4V2, Canada

**Keywords:** saturation correction, fluorescein videoangiography, retina, retinal vascular permeability, arterial input function, gamma-variate function, kinetic tracer modeling

## Abstract

*Objective*. Quantitative retinal fluorescein videoangiography (FVA) can estimate extraction fraction (*E*), but the 8-bit intensity resolution of clinical systems creates a dynamic-range tradeoff: preserving arterial input function (AIF) linearity can restrict tissue signal, particularly for pixel-level analysis. We developed a single-bolus correction method to broaden the usable acquisition range while maintaining *E* estimation accuracy. *Approach*. The method combined truncated-mean reconstruction when sufficient unsaturated arterial pixels remained with gamma-variate interpolation when saturation dominated the first-pass peak. Development simulations used four human-derived AIF templates, empirically characterized signal-dependent noise, and 20 paired realizations evaluated over arterial scaling factors *f* = 0.1–2.25. Tissue curves were generated using a distributed-transit-time AATH model and fitted using a plug-flow AATH model. The locked correction threshold was then evaluated by artificially clipping raw arterial-pixel time curves from 10 originally undersaturated FVAs without adding synthetic noise. Performance was compared with the clipped arterial mean, piecewise cubic Hermite interpolation, and gamma-variate interpolation alone. *Main results*. The combined method completed all 60 patient-by-clipping analyses and produced lower first-pass peak, normalized mean squared error (NMSE), and area-under-the-curve errors than the comparator methods under moderate-to-strong clipping. In kinetic simulations, mean absolute relative ROI *E* error with the uncorrected AIF increased from 17 ± 9% at *f* = 1.25–60 ± 20% at *f* = 2.25, whereas corrected-AIF error remained approximately 10%–20%. Single-pixel *E* error decreased as signal approached the detector ceiling, reached 17 ± 11% at *f* = 1.75, and remained statistically similar across *f* = 0.8–2.0. No single-pixel fits failed at *f* ⩾ 1.0. *Significance*. The correction framework converts modest AIF saturation from a restrictive acquisition limit into a manageable analytical condition. This broader operating range is valuable for 8-bit FVA, supporting acquisition near the detector ceiling to improve tissue signal and enable high-resolution *E* mapping without dual-bolus acquisition.

## 1. Introduction

Indicator-dilution methods have long been used to quantify physiological transport processes from the dynamic passage of an injected tracer through a vascular system (Zierler 2000). In their classical form, such methods estimate parameters such as blood flow, blood volume, and mean transit time from the measured temporal relationship between an arterial input and downstream signal (Meier and Zierler 1954, Zierler 1965, 2000). More recent extensions to diffusible tracers have enabled estimation not only of hemodynamics, but also of exchange between the vascular and extravascular spaces, thereby providing access to parameters related to vascular permeability (St Lawrence and Lee 1998, Vavrek *et al* 2025b). These approaches are attractive because they can provide physiologically interpretable biomarkers from dynamic imaging following a single bolus injection.

Retinal fluorescein videoangiography (FVA) offers a clinically accessible framework in which to apply this type of modeling to the human retina (Tichauer *et al* 2015a, 2015b, Vavrek *et al* 2025b). Using a scanning laser ophthalmoscope (SLO), dynamic fluorescein signal can be measured in retinal arteries and tissue following clinical intravenous sodium fluorescein administration. With an adaptation of the adiabatic approximation to the tissue homogeneity (AATH) model, these data can be used to estimate retinal blood flow and extraction fraction, where extraction fraction reflects the fraction of fluorescein leaving the vasculature on a single pass and is therefore related to retinal vascular permeability (Tichauer *et al* 2015b, Vavrek *et al* 2023). Recent work from our group suggests that this framework may detect elevated retinal vascular permeability even in some individuals with diabetes mellitus but no clinically apparent diabetic retinopathy, raising the possibility that such measurements could serve as an early biomarker of disease progression (Vavrek *et al* 2025b).

A major obstacle to broader quantitative application of this approach is the limited dynamic range of standard clinical FVA systems. Widely used Heidelberg SLO-based acquisitions are effectively stored with 8-bit intensity resolution, limiting the range of fluorescein signals that can be measured linearly within a single study. Quantitative kinetic analysis requires the tissue curve and arterial input function (AIF) to be measured during the same acquisition, but their signal magnitudes differ substantially. Although absorption and scattering in large retinal vessels attenuate the detected arterial fluorescence, the arterial first-pass signal can still exceed the surrounding tissue signal by several times. Acquisition settings that preserve linearity of the AIF may therefore limit tissue signal-to-noise, whereas increasing the detected tissue signal—particularly for high-resolution parametric mapping—risks saturating the AIF (Vavrek *et al* 2025a).

Although substantial AIF saturation was not observed in the human datasets used in the present study, the issue remains important from a quantitative analysis perspective. Because the AIF is a required input for estimation of blood flow and extraction fraction, saturation-induced distortion of the AIF can propagate directly into model bias. In particular, prior analyses indicated that uncorrected oversaturation caused underestimation of extraction fraction and poorer fits to tissue data, which is especially problematic when vascular permeability is itself the parameter of interest (Vavrek *et al* 2025b, 2023). At the same time, stronger fluorescence signal may be advantageous for reducing noise in small tissue ROIs or individual pixels, suggesting a practical tradeoff between avoiding AIF saturation and improving the precision of parametric mapping.

A variety of strategies have been developed in other imaging modalities to address first-pass AIF saturation, particularly in dynamic contrast-enhanced magnetic resonance imaging. These include dual-bolus and dual-sequence methods, as well as reference-region and blind-estimation approaches (Christian *et al* 2004, 2008, Gatehouse *et al* 2004, Groothuis *et al* 2010, Ishida *et al* 2011, Kershaw and Cheng 2011, Li *et al* 2023). However, such approaches are not readily transferable to clinical retinal FVA. Dual-bolus methods increase procedural complexity, require assumptions regarding equivalence between injections, and would likely be difficult to implement robustly in patients who are already required to maintain steady fixation during a bright-light examination. Reference-region and blind-estimation approaches are also challenging in the retina because they rely on assumptions about tissue normality, arrival timing, or scale factors that are not straightforward to satisfy in this setting. These considerations motivate the development of a practical single-bolus correction strategy tailored to clinical FVA.

In this work, a two-part approach for mitigating AIF saturation in retinal FVA was investigated. First, when only a subset of pixels within an arterial region of interest is clipped, the ROI mean is corrected using a censored-mean reconstruction method. Second, when clipping dominates the arterial ROI during the first pass, the missing portion of the AIF is reconstructed by fitting a simplified gamma-variate function (GVF), motivated by the well-established resemblance between first-pass indicator-dilution curves and gamma-variate behavior (Davenport 1983, Amartey *et al* 2024). Because substantial AIF saturation was absent in the available human datasets, this strategy was evaluated primarily using clinically realistic simulations derived from human FVA data. The specific objective was to determine whether this single-bolus saturation-correction framework could preserve accurate estimation of extraction fraction under clinically relevant levels of AIF saturation, thereby enabling future acquisition strategies designed to improve high-resolution parametric mapping, particularly at the single-pixel level.

## 2. Methods and materials

### 2.1. Human retinal FVA data collection

Retinal FVA data were acquired at the Department of Ophthalmology and Visual Sciences, University of Illinois Chicago, and the Department of Ophthalmology, Northwestern University, from a larger cohort of consenting participants enrolled under institutional review board approval. All procedures were conducted in accordance with the Declaration of Helsinki, and written informed consent was obtained from all participants. FVA was performed using a SLO (SPECTRALIS HRA + OCT, Heidelberg Engineering, Franklin, MA, USA) equipped with a 55-degree widefield lens. Either the right or left eye was imaged in High Speed mode with manual sensitivity set to 74%. Immediately after scan initiation, a 0.5 ml intravenous bolus of 10% sodium fluorescein (Fluorescite, Alcon Laboratories, Fort Worth, TX, USA) was administered, followed by an approximately 2 ml saline flush; dynamic imaging continued for approximately 2 min. Four nonsaturated AIFs were selected as simulation-development templates. A separate set of 10 originally undersaturated FVAs was used for empirical raw-pixel validation. The empirical validation cohort comprised four controls, three participants with diabetes mellitus without clinically apparent diabetic retinopathy (DMnoDR), and three participants with mild nonproliferative diabetic retinopathy (NPDR). Mean age at imaging was 34 ± 7, 40 ± 10, and 60 ± 10 years, respectively; the overall sex distribution was seven male and three female. The DMnoDR group included two participants with type 1 and one with type 2 diabetes, whereas all three participants with mild NPDR had type 2 diabetes. Mean diabetes duration was 10 ± 10 years in the DMnoDR group and 8 ± 10 years in the mild NPDR group, with corresponding mean HbA1c values of 6.4 ± 0.7% and 6.1 ± 0.5%. Additionally, six FVAs were selected purposively to illustrate one undersaturated and one visibly oversaturated example from each diagnostic group (control, DMnoDR, and mild NPDR). These six cases were neither matched nor used for inferential testing or ground-truth validation. The six FVAs were obtained from four male and two female subjects. The control, DMnoDR, and mild NPDR groups’ respective mean age at imaging was 50 ± 20 years, 44 ± 6 years, and 60 ± 10 years. The DMnoDR and mild NPDR subjects respective mean diabetes duration was 4 ± 2 years and 10 ± 10 years; all subjects from both DMnoDR and mild NPDR groups had type 2 diabetes.

### 2.2. Human data preprocessing and region-of-interest selection

All FVAs underwent removal of poor-quality frames and motion correction following previously described methods (O’Connor *et al* 1998, Vavrek *et al* 2023, 2025b). Frames with severe blink artifact, loss of retinal visibility, or obvious registration failure were excluded, and residual eye motion was corrected before ROI analysis. Saturated arterial pixels were identified in the raw 8-bit data at the detector ceiling of 255 arbitrary units (a.u.) and retained as censored observations for the correction procedure; saturation was not treated as a frame-removal operation. Background was subsequently subtracted. Therefore, a raw saturation plateau of 255 a.u. appears at approximately 255 a.u. minus the sequence-specific baseline in the background-subtracted curves. Tissue ROIs were selected to avoid visibly saturated pixels.

For each included FVA, one retinal arterial ROI and one nearby retinal tissue ROI were manually selected. The arterial ROI was drawn over a visibly well-defined retinal artery. The tissue ROI was selected in adjacent retinal parenchyma to minimize large-vessel contamination while maintaining proximity to the sampled arterial input. The mean fluorescence signal within the arterial ROI was defined as the measured AIF, *S*_a_(*t*), and the mean fluorescence signal within the tissue ROI was defined as the tissue curve, *S*_tis_(*t*). Here, *S*_a_(*tₙ*) and S_tis_(*tₙ*) denote discrete, background-subtracted ROI-mean time-activity curves sampled at the retained acquisition times *tₙ*; no analytic functional form was assumed for either measured curve at this stage. Isolated missing frames caused by image-quality rejection were filled using piecewise cubic Hermite interpolation (PCHIP). This preprocessing interpolation was distinct from the saturation reconstruction: the latter used the truncated-mean correction when sufficient unsaturated arterial pixels remained and a gamma-variate first-pass fit when the arterial ROI was predominantly saturated. Background was estimated as the mean signal over the first five pre-injection frames and subtracted from each curve.

### 2.3. Selection of human AIFs for simulations

To construct clinically realistic simulations, four non-saturated AIFs extracted from human retinal FVA datasets were used as simulation templates. These included one healthy control, two participants with DMnoDR, and one participant with mild NPDR, corresponding to the representative curves shown in figure 1(A). Each template AIF was background-subtracted and smoothed before use in simulation. For each simulated dataset, one of the four template AIFs was selected at random.

**Figure 1.**
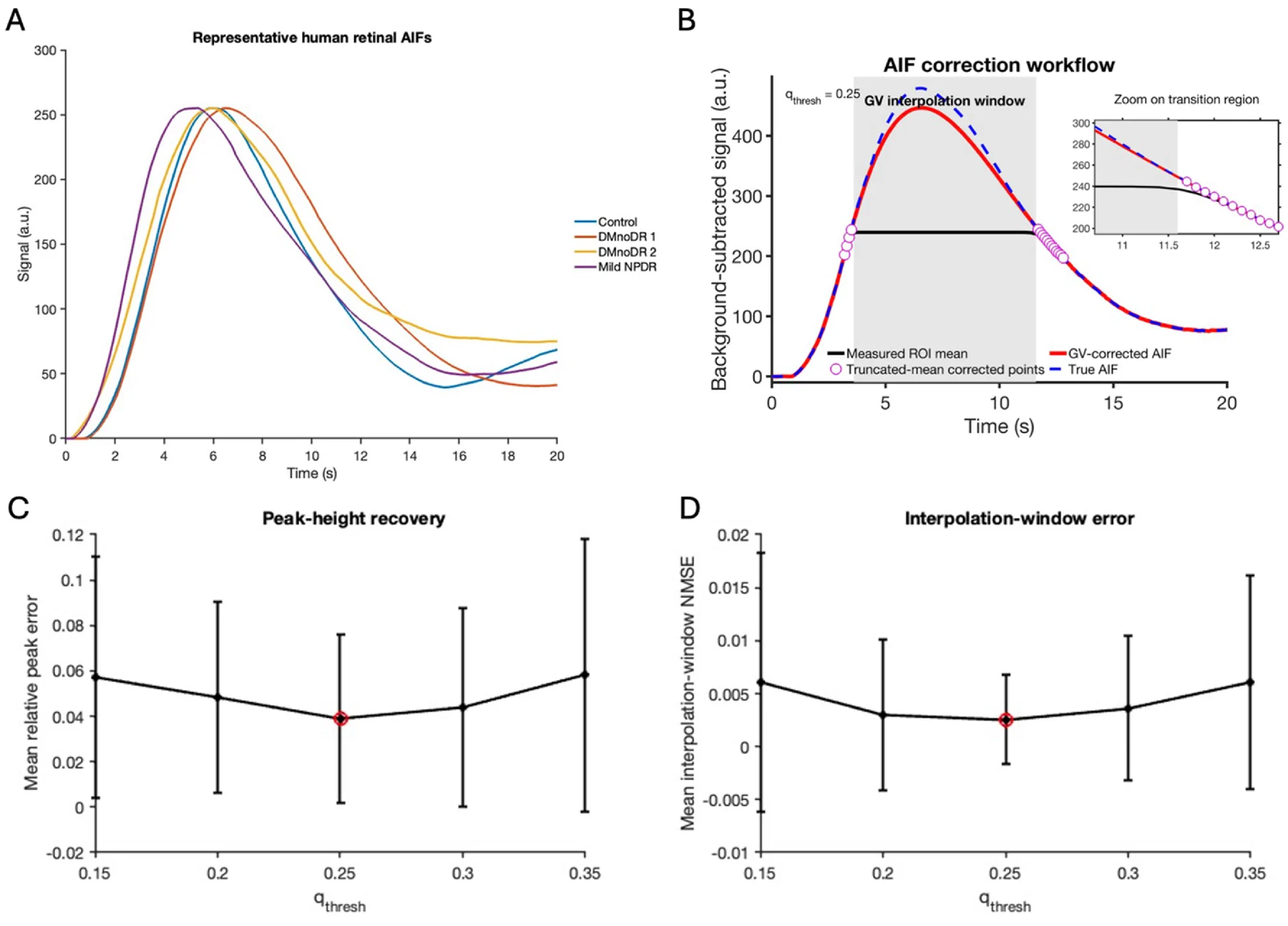
Human-derived arterial input functions (AIFs), example saturation correction, and development of the correction threshold. (A) Four human FVA AIF templates. (B) Example simulated correction; truncated-mean-corrected points are shown as open magenta markers above the fitted curve, and the inset shows the right transition. (C) Mean absolute relative peak-height error, |*P*_corr_—*P*_true_|/|*P*_true_|, as a function of *q*_thresh_. (D) Mean first-pass interpolation-window NMSE. The development-selected operating value *q*_thresh_ = 0.25 is marked in red; it was locked before independent empirical validation and is not interpreted as a universal optimum.

The four templates were used only for simulation development and threshold selection. The resulting operating threshold (*q*_thresh_ = 0.25) and fitting-window procedure were locked before application to the independent 10-FVA raw-pixel validation cohort. The threshold is treated as a prespecified operating value, not as a universal optimum.

### 2.4. Noise characterization

Noise characteristics were estimated from undersaturated human FVA data acquired on the Heidelberg SLO, following Herberich *et al* (2012). Residuals were obtained after temporal smoothing and evaluated as functions of local mean intensity. Only samples with mean signal ⩽120 a.u. were used for fitting because higher signals showed deviations compatible with incipient saturation and other nonlinearities. Over the fitted range, residual variance increased approximately linearly with mean signal and residual standard deviation followed an approximately square-root dependence. The simulation therefore added independent Gaussian noise with standard deviation sigma(*S*) = sqrt(max(*S*,0)) before 8-bit quantization. Applying this relation above 120 a.u. is an extrapolation used only in the synthetic experiment; the independent raw-pixel clipping experiment added no synthetic noise and therefore did not depend on this extrapolation.

### 2.5. Simulation framework

#### 2.5.1. Arterial signal and pixel generation

Each human-derived arterial template *A*(*t*) was normalized to its maximum and scaled to an 8-bit arterial dynamic range. For an oversaturation factor *f* and arterial background *B*_a_, the noise-free arterial signal was

\begin{equation*}{S_{{\mathrm{a}},0}}\left( t \right) = \frac{{f\left( {255 - {B_{\mathrm{a}}}} \right)}}{{\max \left[ {A\left( t \right)} \right]}} + {B_{\mathrm{a}}}.\end{equation*}

For each realization, an arterial ROI pixel count *M*_a_ was drawn as specified in table 1. Independent signal-dependent Gaussian perturbations were then applied to *S*_a_*,_0_(t)* for each pixel, after which values were quantized and clipped to the 8-bit interval [0, 255]. The measured arterial ROI curve was calculated from the resulting pixel ensemble and processed by the same saturation-correction algorithm used for human data.

**Table 1.** Physiological, optical, and acquisition parameters used in the simulations.

Symbol	Definition (units)	Sampling rule or calculation	Source/constraint
*F*	Blood flow (ml min^−1^ (100 g)^−1^)	Normal (50, 7)	(Kaufman and Albert 2003)
*E*	Extraction fraction (unitless)	Uniform (0.01, 0.2)	(Vavrek *et al* 2025b)
ζ	Tissue-to-artery detection-efficiency ratio (unitless)	Uniform (12, 19)	(Vavrek *et al* 2025b)
*t*_a_	Artery-to-tissue arrival time (s)	Uniform (0, 2)	(Vavrek *et al* 2025b)
${\bar t_t}$	Mean vascular transit time (s)	Calculated using the Grubb Relationship assuming blood volume is approximately equal to 0.88*F*^0.3^	(Ito *et al* 2001)
*V_e_*	Retinal tissue-to-plasma distribution volume (ml (100 g)^−1^)	Fixed at 50	(Rohrbach *et al* 2017)
*k*_adb_	Back-diffusion rate constant (s^−1^)	*EF*/*V_e_*/(60 s min^−1^)	(St. Lawrence and Lee 1998)
ρ	Tissue density factor (g ml^−1^)	Fixed at 0.97	(Rohrbach *et al* 2017)
α	Gamma transit-time distribution shape (unitless)	Normal (30, 5)	(Hickam and Frayser 1965; St Lawrence *et al* 2013)
*M*_a_	Arterial ROI pixel count (# of pixels)	Normal (300, 100)	(Vavrek *et al* 2025b)
*M*_t_	Tissue ROI pixel count (# of pixels)	Normal (1800, 600)	(Vavrek *et al* 2025b)
*B*_a_	Arterial background (intensity units)	Normal (10, 4)	(Vavrek *et al* 2025b)
*B*_t_	Tissue background (intensity units)	Normal (12, 4)	(Vavrek *et al* 2025b)

Normal (*μ, σ*) denotes a normal distribution with mean *μ* and standard deviation *σ*; Uniform (*a, b*) denotes a continuous uniform distribution bounded by *a* and *b*. Arterial and tissue ROI pixel-count draws were rounded to positive integers.

The simulation retained the 0.1 s interpolated time grid of the selected human AIF template. Template-dependent durations were approximately 40–55 s (approximately 400–550 samples). Arterial and tissue ROI pixel counts were sampled according to table 1; positive integer draws had means (standard deviations) of 300 (100) and 1800 (600) pixels, respectively.

#### 2.5.2. Tissue generation with a distributed-transit-time AATH model

For each simulated background-free AIF (*S*_a*,0*_(*t*)), corresponding noise-free tissue curves (*S*_tis,0_(*t_n_*)) were generated using the distributed-transit-time form of the adiabatic approximation to the tissue-homogeneity model, including vascular dispersion (St Lawrence *et al* 2013) that incorporated an explicit arterial-to-tissue arrival delay (Tichauer *et al* 2015a). Briefly, this and similar models assume that the tissue can be approximated as a linear time-invariant system such that the concentration of the imaging agent, *Q*(*t*), is equivalent to a blood flow (*F*)-scaled convolution of the arterial input concentration of the imaging agent, *C*_a_(*t*), and an impulse residue function, *R*(*t*).

\begin{equation*}Q\left( t \right) = F{C_{\mathrm{a}}}\left( t \right) * R\left( t \right).\end{equation*}

The distributed-transit time AATH assumes the following functional form for *R*(*s*), where *s* is the elapsed time after a tissue arrival delay *t*_a_:

\begin{align*} {R_{{\mathrm{AATH}_{\mathrm{VD}}}}}\left( t \right) = 1-\int_0^t \frac{{t^{\alpha -1}}{\mathrm{e}^{\frac t{\tau}}}}{{\tau^\alpha}\Gamma \left( \alpha \right)}\mathrm{d} t+E\cdot {\mathrm{e}^{-{k_{\mathrm{adb}}}t}}\int_0^t \frac{{t^{\alpha -1}}{\mathrm{e}^{\frac t{\tau}}}}{{\tau^\alpha}\Gamma \left( \alpha \right)}{\mathrm{e}^{{k_{\mathrm{adb}}}t}}\mathrm{d} t, \end{align*}

where *α* is a unitless parameter related to the width of the distribution in vascular transit times, $\tau = {t_\tau}/\alpha$, where ${t_\tau}$ represents the mean vascular transit time, and ${\Gamma}\left( \alpha \right)$ represents the gamma function: $\Gamma \left( \alpha \right) = \int_0^\infty {t^{\alpha -1}}{\mathrm{e}^{-t}}\mathrm{d} t$. The parameters *E* (extraction fraction) and *k*_adb_ (adiabatic rate constant) are associated with vascular permeability; *E* represents the fraction of fluorescein that will extravasate in an ROI on a single pass through the ROI and *k*_adb_ represents a rate constant for the likelihood of diffusion of fluorescein from the extravascular extracellular space into the vascular space. The parameter *k*_adb_ was the rate constant governing transport of the imaging agent from retinal tissue back to blood (or ‘back-diffusion rate constant’), *u* was a dummy variable for time, ${\bar t_t}$ was the mean vascular transit time, Γ represented the Gamma function, and α was a factor related to the Gamma transit-time distribution (St Lawrence *et al* 2013). The noise-free tissue signal was generated by discrete convolution:

\begin{equation*}{S_{{\mathrm{tis}},0}}\left( {{t_n}} \right) = {B_{\mathrm{t}}} + \frac{{\rho F\zeta }}{{6000}}{{\Delta }}t\mathop {\mathop \sum \nolimits }\limits_m {C_a}\left( {{t_m}} \right){R_{{\mathrm{dtt}} - {\mathrm{AATH}}}}\left( {{t_n} - {t_m}} \right)\end{equation*}

where *B*_t_ was a tissue background signal, *ρ* was the tissue-density factor, *F* was physical blood flow (in units of ml min^−1^ (100 g)^−1^—standard units used clinically), the 6000 (60 s min^−1^ 100 g (100 g)^−1^) was a scaling factor to convert *F* to units of s^−1^, and *ζ* was the tissue-to-artery optical detection-efficiency ratio. The ranges of the model parameters that were sampled in all simulations are summarized in table 1.

Clinically realistic simulations were carried out to evaluate the effect of AIF saturation on kinetic-parameter estimation and on the performance of the proposed correction method. Twenty Monte Carlo realizations were generated. Each realization used one randomly selected human-derived AIF template and one draw of the physiological parameters, optical scaling, ROI sizes, arterial background *B*_a_, and tissue background *B*_t_ specified in table 1. A paired design was used across saturation levels: i.e. the same realization (including its template, parameters, backgrounds, ROI sizes, and underlying standardized noise draws) was evaluated at every *f*. Only the imposed signal scale and the resulting signal-dependent noise, 8-bit quantization, and clipping changed with *f*. Saturation levels ranged from *f* = 0.1–2.25, where *f* = 1 denotes a noise-free arterial peak at the nominal saturation threshold.

To emulate arterial ROI measurements, the scaled AIF was used to generate a population of arterial pixel time courses with signal-dependent noise and 8-bit quantization. The measured arterial ROI mean was then obtained by averaging across pixels at each time point. Corresponding tissue curves were generated using an AATH model that included vascular dispersion distributed as a GVF and an arterial-to-tissue arrival-time offset (Koh *et al* 2001, Schabel 2012, Tichauer *et al* 2015b). For each arterial realization, one tissue ROI realization and one single-pixel tissue realization were retained for downstream fitting.

### 2.6. Saturated-mean correction

Partial saturation causes the directly measured arterial ROI mean to underestimate the underlying mean pixel intensity. To correct this bias, a truncated-normal moment reconstruction was applied when some, but not all, arterial ROI pixels reached the detector ceiling (Johnson *et al* 1994). All saturation classification and reconstruction calculations were performed using the raw 8-bit pixel values before background subtraction. Let *M* pixels in an ROI at time point *n* be represented by:

\begin{equation*}{X_n} = \left\{ {x_{1,n}},{x_{2,n}},\ldots ,{x_{M,n}} \right\}\end{equation*}

then the set of unsaturated pixels can be represented as:

\begin{equation*}{U_n} = \left\{ {{x_{i,n}} \in {X_n}:{x_{i,n}} &lt; {x_{{\mathrm{sat}}}}} \right\}.\end{equation*}

With *x*_sat_ = 255 a.u. The fraction of unsaturated pixels is then

\begin{equation*}{q_n} = \frac{\left| {U_n} \right|}{M}.\end{equation*}

If *q_n_* = 1, the uncorrected ROI mean was retained. If *q_n_* exceeded a minimum reliability threshold (empirically, *q_n_* ⩾ 0.25), the underlying pixel-intensity distribution was modeled as Gaussian with a mean of *μ_n_* and a standard deviation of *σ_n_*. Under this assumption correction of the saturated mean acted as a right-truncation of the distribution at *x*_sat_. Specifically, if the sample mean and population standard deviation (i.e. using *M* in the denominator) of the uncensored pixels, *U_n_*, is represented by ${\bar x_{{U_n}}}$ and ${s_{{U_n}}},$ respectively, the standard normal quantile associated with the unsaturated fraction is represented by the parameter *α_n_* = *Φ*^−1^(*q_n_*)—where *Φ*^−1^(*^.^*) represents the inverse cumulative distribution function of the standard normal distribution—and the corresponding standard normal density is represented by *φ_n_* = *φ*(*α_n_*), then the so-called Mills ratio can be defined as

\begin{equation*}{\lambda_n} = \frac{{\phi_n}}{{q_n}}\end{equation*}

the estimated standard deviation of the underlying population can be approximated by

\begin{equation*}{\hat{\sigma}_n} = \frac{{s_{{U_n}}}}{\sqrt{1-{\alpha_n}{\lambda_n}-{\lambda_n^2}}}\end{equation*}

and then and estimated saturation-corrected mean can be approximated as

\begin{equation*}{\hat \mu _n} = {\bar x_{{U_n}}} + {\hat \sigma _n}{\lambda _n}.\end{equation*}

### 2.7. Gamma-variate interpolation of saturation-dominated arterial time points

Time points for which *q_n_* < *q*_thresh_ occurred during the first-pass arterial peak and were reconstructed using a simplified GVF (*G*(*t*)):

\begin{equation*}G\left( t \right) = K{\left( {t - {t_0}} \right)^p}{{\mathrm{e}}^{ - b\left( {t - {t_0}} \right)}},t &gt; {t_0}.\end{equation*}

The gamma-variate parameters [*K, p, b, t*_0_] were initialized at [30, 1, 1, 1] with lower bounds [0, 0, 0, −10] and upper bounds [400, 18, 10, 10]. Bounded nonlinear least squares was implemented with MATLAB lsqcurvefit using the Levenberg–Marquardt option, tolerances of 10^−12^, and maximum iteration and function-evaluation counts of 100 000. Candidate left extensions were 5:5:60 samples and candidate right extensions were 5:5:65 samples (0.5–6.0 s and 0.5–6.5 s on the 0.1 s simulation grid). Each candidate used every nonmissing measured or truncated-mean-corrected sample in the window and required at least four contributing samples.

Candidate windows were ranked by unweighted mean squared residual plus a small window-length penalty, 10^−4^(*L*_eff_ + *R*_eff_)—where *L*_eff_ and *R*_eff_ were effective number of points to the left and right of the interpolation window, respectively—and a penalty of 0.01 when a fitted parameter was at a bound. Candidates that failed or returned nonfinite values were discarded. The selected gamma-variate values replaced only samples designated missing; measured and truncated-mean-reconstructed samples were retained. No value or derivative continuity constraint was imposed at the joins, so value and slope mismatch were evaluated explicitly in the empirical validation.

Threshold selection used absolute relative peak-height error, |*P*_corr_—*P*_ref_|/|*P*_ref_|—where *P*_corr_ was the peak of the fitted gamma-variate and *P*_ref_ was that of the scaled raw data peak—as the primary development criterion, with first-pass NMSE as a secondary diagnostic. The selected *q*_thesh_ = 0.25 was then locked. In the independent empirical cohort, *q*_thresh_ values of 0.20, 0.25, and 0.30 were examined only as a prespecified sensitivity analysis and were not used to select a new threshold.

### 2.8. Empirical arterial-pixel validation

To evaluate the correction framework under the spatial heterogeneity present in measured retinal arteries, an independent empirical validation was performed using arterial-pixel time–activity curves from one originally undersaturated arterial ROI in each of 10 human FVAs (Vavrek *et al* 2025b). The native arterial ROIs contained 23–148 pixels (median, 57 pixels; mean ± SD, 78 ± 43 pixels). These native ROI sizes were retained to preserve the participant- and acquisition-level variability present in the measured arterial data. For each ROI, the original raw 8-bit pixel intensities were reconstructed by adding the measured pixel-specific background to the corresponding background-subtracted pixel time-activity curves. All native pixel values were below the detector ceiling. The original unclipped arterial ROI mean was retained as the empirical reference curve; because it contained measured noise and other acquisition effects, it was not considered noise-free ground truth. No synthetic noise was added during this experiment.

Artificial saturation was imposed directly on the raw arterial-pixel intensities at target oversaturation factors of *f* = 1, 1.25, 1.5, 1.75, 2, and 2.25. For each factor, the artificial integer-valued detector ceiling was defined as

\begin{equation*}{c_f} = {\mathrm{round}}\left[ {B + \frac{{P - B}}{f}} \right]\end{equation*}

where *B* was the mean pre-injection pixel background and *P* was the raw-scale first-pass peak of the original arterial ROI mean. Pixel intensities exceeding *c_f_* were set equal to *c_f_*, after which the arterial ROI curve was reconstructed using the same saturation-correction framework described in sections 2.6 and 2.7. Analyses were restricted to the first 20 s. The unsaturated-pixel threshold was fixed at the simulation-derived value *q*_thresh_ = 0.25 and was not re-optimized using the empirical validation cohort.

The proposed truncated-mean plus gamma-variate reconstruction was compared with the clipped arterial ROI mean (no correction), the mean of the remaining unsaturated pixels, truncated-mean correction alone, PCHIP interpolation of the truncated-mean curve, and gamma-variate interpolation without truncated-mean points. The original unclipped measured ROI mean served as an empirical oracle reference, not as noise-free ground truth. Incomplete curves were counted as method failures. Metrics included signed and absolute relative peak error, first-pass area-on-the-curve (AUC) error, first-pass NMSE, absolute arrival-time error, time-to-peak error, full width half maximum (FWHM) error, and value and first-derivative mismatch at both interpolation joins. Cohort means and 95% confidence intervals were estimated from 2000 participant-level bootstrap resamples. The prespecified *q*_thresh_ sensitivity analysis used 0.20, 0.25, and 0.30.

### 2.9. Kinetic parameter estimation

To avoid an exact generator-estimator match, tissue curves generated with the distributed-transit-time AATH model were analyzed with the retinal FVA-adapted plug-flow AATH model described previously (Vavrek *et al* 2025b). The measured tissue fluorescence signal *S*_tis_(*t*) was modeled as the convolution of the measured arterial signal *S*_a_(*t*) with an arrival-time-adjusted AATH residue *R*_AATH_(*t*−*t_a_*) (St. Lawrence and Lee 1998):

\begin{equation*}{S_{{\mathrm{tis}}}}\left( t \right) = \zeta F{S_{\mathrm{a}}}\left( t \right) * {R_{{\mathrm{AATH}}}}\left( {t - {t_{\mathrm{a}}}} \right)\end{equation*}

\begin{equation*}{R_{\mathrm{AATH}}}\left( t \right) = \left\{ \begin{array}{c} 1,\;\;\;\;\;\;\;0 \le t \le {\bar t_t} \\ E{\mathrm{e}^{-{k_{\mathrm{adb}}}\left( t-{\bar t_t} \right)}},\;\;t &gt; {\bar t_t} \end{array} \right..\end{equation*}

Parameter estimation used the retinal FVA-adapted plug-flow AATH model and bounded Nelder–Mead optimization (fminsearchbnd) in MATLAB. The fitted parameter order was [ζF, *t*_a_, ${\bar t_t}$, *E, k*_adb_]. Nominal initial values were [10, 1, 2, 0.10, 0.001], with lower bounds [2, 0, 0, 0.001, 10^−6^] and upper bounds [6000, 8, 10, 0.40, 0.1]. The first stage estimated ζF, *t*_a_, and ${\bar t_t}$ with a boxcar residue over the rising portion of the tissue curve. The second stage fit all five parameters over the full curve from five dispersed starting vectors and retained the solution with the smallest residual sum of squares (Vavrek *et al* 2025b). The search used maximum counts of 2500 iterations and 5000 function evaluations, with MATLAB options of TolX = 10^−6^ and TolFun = 10^−8^.

For each simulated realization, ROI-tissue fits used the true unsaturated AIF, the raw arterial ROI mean, and the corrected arterial ROI mean. Single-pixel tissue fits used the corrected AIF to evaluate the high-resolution use case. The raw-AIF/single-pixel combination was not used to support the single-pixel claim because it superimposes severe AIF clipping and single-pixel tissue noise rather than isolating correction performance.

### 2.10. Performance metrics and analysis

Extraction fraction estimates were compared with their known generating values. The primary displayed metric was absolute relative error, 100|*E*_fit_—*E*_true_|/*E*_true_. Signed bias, mean absolute error, root-mean-square error, fit NMSE, and fit-failure rate were also calculated. A fit was flagged when the optimizer did not return a finite solution or fit NMSE exceeded 0.20; no returned parameter estimates were removed as outliers. The same metrics were calculated for *k*_adb_. Points in all figures are means and error bars are 95% confidence intervals across the 20 paired realizations.

## 3. Results

### 3.1. Simulation study

The simulation study was designed to evaluate the effect of AIF saturation on extraction fraction estimation and to test whether the proposed correction framework could preserve estimation accuracy under higher-signal conditions. Twenty paired realizations were generated. One of the four human-derived AIF templates shown in figure 1(A) was selected once for each realization, and the same simulated physiological and acquisition case was then evaluated across all arterial oversaturation levels.

Figure 1 summarizes development of the AIF correction framework using the four simulation templates. Absolute relative peak-height error was the primary selection metric and first-pass NMSE was secondary. The value *q*_thresh_ = 0.25 was selected from these development simulations and then locked before the independent raw-pixel validation. The empirical sensitivity analysis over *q*_thresh_ = 0.2-0.3 did not identify a single threshold that was best at every clipping level; 0.25 is therefore treated as a prespecified operating choice rather than a universally optimal value.

The signal-dependent noise behavior used in the simulations is summarized in figure 2. Residual variance increased approximately linearly with mean signal over the non-saturated range analyzed (slope = 0.5, intercept = −2.9), while residual standard deviation followed an approximately square-root dependence on signal intensity. To avoid bias from saturation-related nonlinearity at higher signal levels, model fitting was restricted to data with mean signal ⩽120 arbitrary units. These empirically derived relationships were then incorporated into the arterial and tissue simulations.

**Figure 2.**
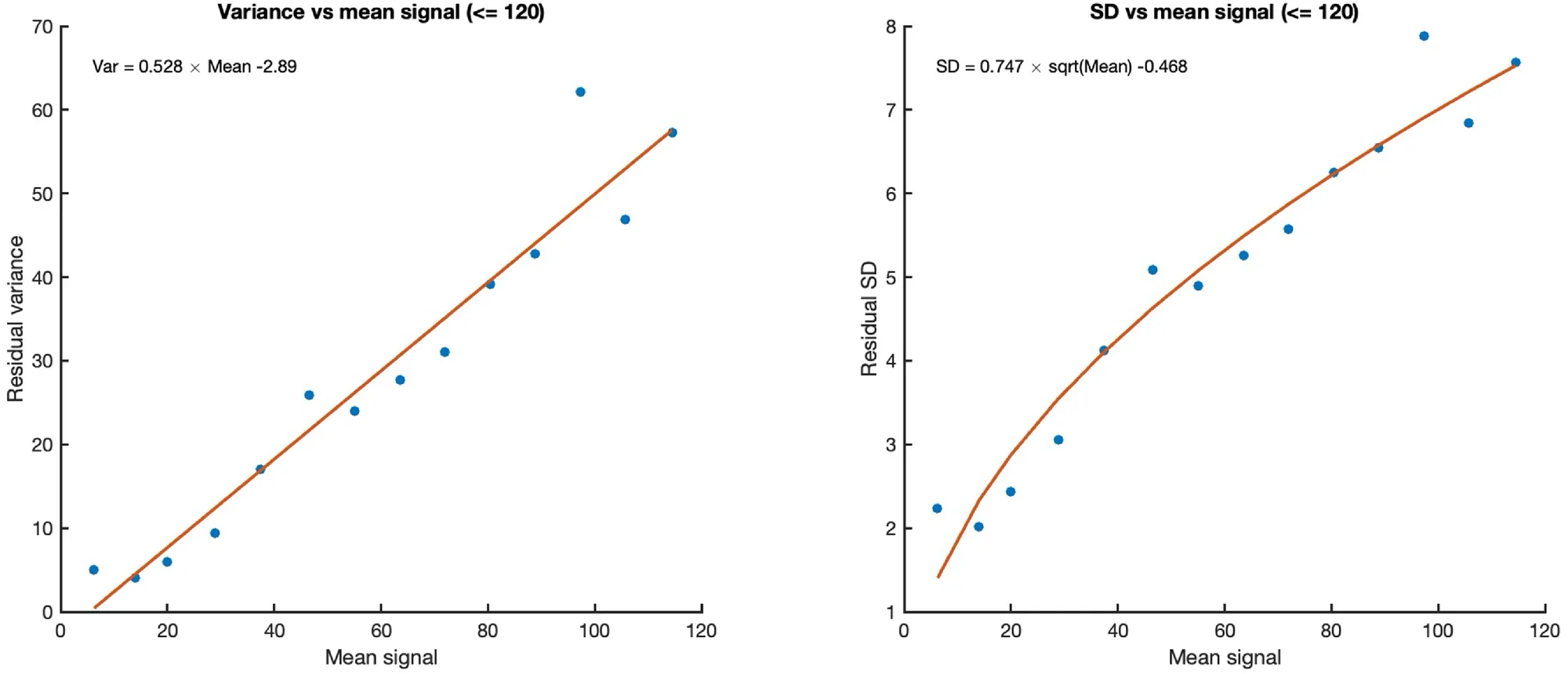
Empirical characterization of signal-dependent noise in undersaturated Heidelberg SLO fluorescein videoangiography data. Residual variance (left) and residual standard deviation (right) are plotted as functions of mean signal intensity after temporal smoothing of the image sequence. To reduce bias from incipient saturation and other high-signal nonlinearities, only data with mean signal ⩽120 arbitrary units were included in the fitted noise models. Over this range, variance increased approximately linearly with mean signal, and standard deviation followed an approximately square-root dependence on mean signal. These empirically derived relationships were used to generate noisy arterial and tissue signals for the simulation study.

Examples of corrected AIFs are shown in figure 3 for representative control, DMnoDR, and mild NPDR template shapes across increasing arterial oversaturation levels. Below saturation, the measured, corrected, and true AIFs were nearly indistinguishable. As oversaturation increased above the nominal 8-bit limit, the measured arterial peak became increasingly distorted, whereas the corrected AIF remained in close agreement with the underlying true curve across the first-pass region. These examples illustrate that the combined truncated-mean and gamma-variate approach was able to recover the major first-pass AIF features over the saturation range studied.

**Figure 3.**
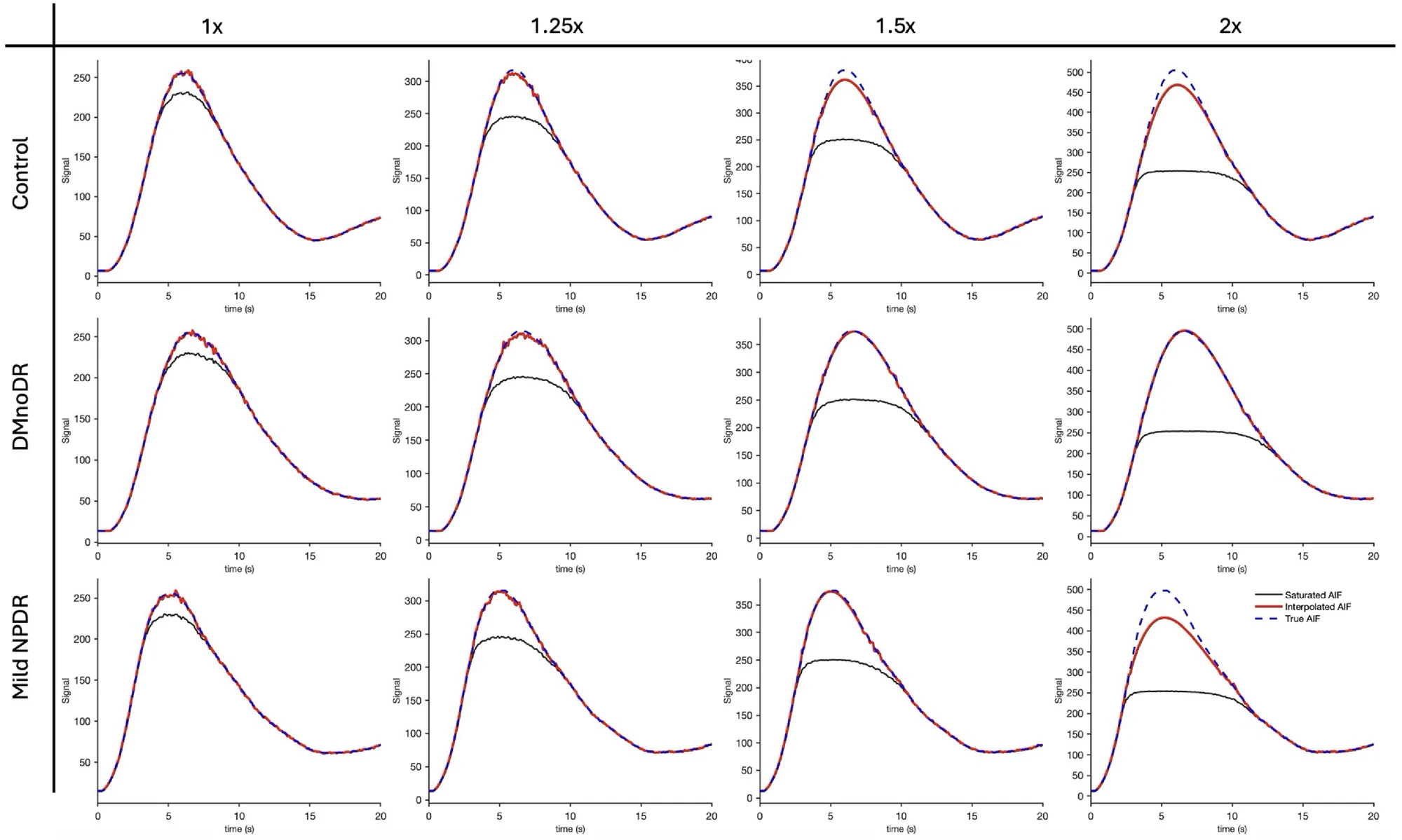
Illustrative mean arterial input function (AIF) corrections across human-derived templates and saturation levels. Each displayed curve and shaded band summarizes five noise realizations (mean and 1 standard deviation) used only to visualize typical curve behavior. The quantitative parameter-error analysis in figure 5 used the separate set of 20 paired realizations described in Methods. Black, red, and blue dashed curves show the measured saturated, corrected, and true AIFs, respectively; *q*_thresh_ = 0.25 was used throughout.

The locked correction framework was next evaluated using the raw arterial-pixel time–activity curves from 10 independent, originally undersaturated human FVAs (figure 4). Artificial clipping was applied directly to the measured 8-bit pixel intensities without adding synthetic noise, and the original unclipped arterial ROI mean was used as the empirical reference. At the locked threshold of *q*_thresh_ = 0.25, the proposed truncated-mean plus gamma-variate method completed all 60 patient-by-clipping analyses without an optimizer failure or PCHIP fallback.

**Figure 4.**
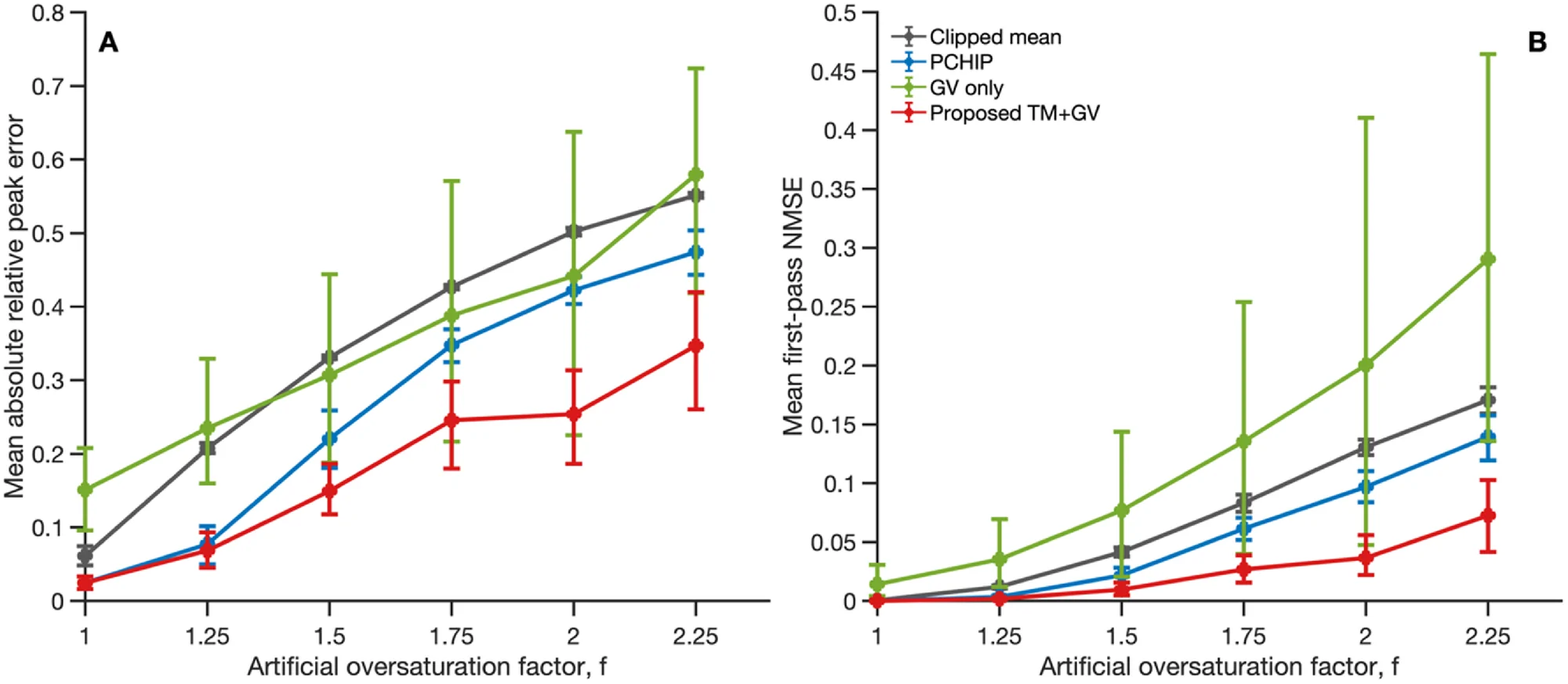
Empirical validation of arterial input function saturation correction using measured arterial-pixel time–activity curves. Artificial detector clipping was applied to raw arterial-pixel intensities from 10 originally undersaturated human fluorescein videoangiographies at target oversaturation factors (*f* = 1–2.25). No synthetic noise was added, and the original unclipped arterial ROI mean was used as the empirical reference rather than as noise-free ground truth. Reconstruction was performed using the independently selected threshold (*q*_thresh_ = 0.25). (A) Mean absolute relative error in recovered first-pass peak height. (B) Mean normalized mean squared error (NMSE) over the first 20 s. Methods shown are the uncorrected clipped ROI mean (gray), piecewise cubic Hermite interpolation of the truncated-mean curve (PCHIP; blue), gamma-variate interpolation without truncated-mean-corrected points (GV only; green), and the proposed combined truncated-mean plus gamma-variate reconstruction (TM + GV; red). Points represent cohort means and error bars represent participant-level 95% bootstrap confidence intervals from 2000 resamples.

The proposed method had lower mean absolute relative peak error, first-pass NMSE, and first-pass AUC error than the clipped ROI mean or PCHIP under moderate-to-strong clipping. Values are reported as mean ± SD using the available finite participant-level measurements. At *f* = 1.5, absolute relative peak error was 0.15 ± 0.06 for the proposed method, compared with 0.22 ± 0.07 for PCHIP and 0.331 ± 0.004 for the clipped mean; absolute AUC errors were 0.04 ± 0.03, 0.07 ± 0.02, and 0.12 ± 0.02, respectively. At *f* = 2, the corresponding peak errors were 0.3 ± 0.1, 0.42 ± 0.03, and 0.502 ± 0.009, and AUC errors were 0.11 ± 0.05, 0.20 ± 0.03, and 0.25 ± 0.03. Across *f* = 1–2.25, mean absolute arrival-time error did not exceed 0.3 ± 0.4 s, mean absolute time-to-peak error did not exceed 2 ± 4 s, and mean absolute FWHM error did not exceed 3 ± 2 s (*n* = 8 at *f* = 2.25). For joins present at *f* = 1.25–2.25, mean relative value mismatch did not exceed 0.03 ± 0.02 and mean relative slope mismatch did not exceed 0.4 ± 0.3 s^−1^. Residual error nevertheless increased with clipping severity, reaching a mean absolute relative peak error of 0.4 ± 0.1 at *f* = 2.25.

The principal downstream results are shown in figure 5. Values are reported as mean ± SD across the 20 paired realizations unless otherwise indicated. With the raw AIF and an ROI tissue curve, mean absolute relative *E* error increased from 17 ± 9% at *f* = 1.25–60 ± 20% at *f* = 2.25. With the corrected AIF, ROI error ranged from 10 ± 10% at *f* = 1.75–20 ± 10% at *f* = 2.25. Across *f* = 1.25–2.25, corrected-AIF ROI fits had mean signed *E* bias from 0 ± 0.01–0.01 ± 0.02, mean absolute error from 0.008 ± 0.007–0.02 ± 0.02, and RMSE from 0.01 to 0.03. Single-pixel error decreased to a broad minimum near the nominal threshold and modest oversaturation. The lowest mean single-pixel error was 17 ± 11% at *f* = 1.75, but confidence intervals overlapped across *f* = 0.8–2.0; no unique optimum was inferred. Single-pixel fit-failure rates were 100%, 85%, and 5% at *f* = 0.1, 0.5, and 0.8, respectively, and 0% at *f* ⩾ 1.0.

**Figure 5.**
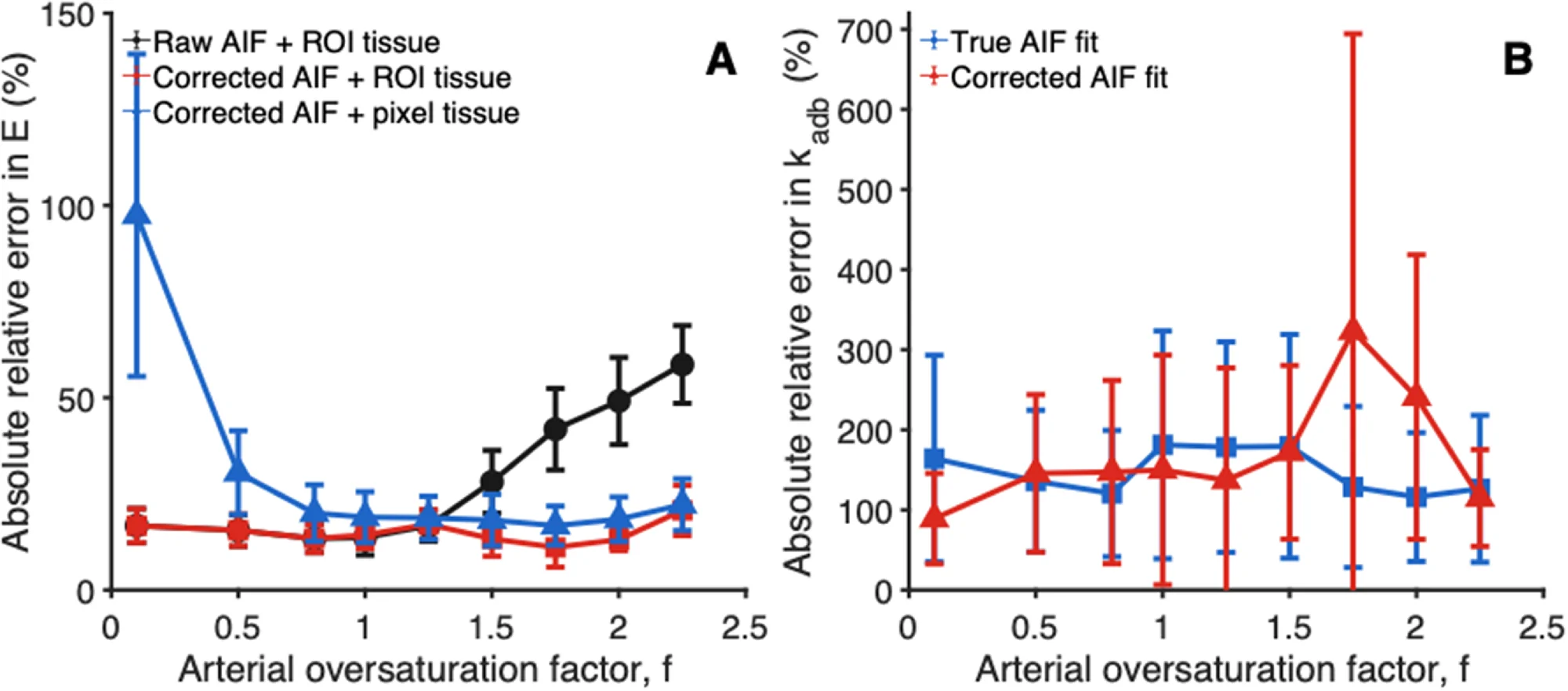
Downstream kinetic-parameter sensitivity to arterial input function (AIF) saturation and correction. (A) Mean absolute relative error in extraction fraction (*E*), expressed as a percentage of the generating E value, for the raw AIF with a tissue region-of-interest (ROI) curve (black), the corrected AIF with a tissue ROI curve (red), and the corrected AIF with a single-pixel tissue curve (blue). (B) Mean absolute relative error in the back-diffusion rate constant *k*_adb_ when each ROI tissue curve was fitted using the true unsaturated AIF (blue) or the gamma-variate-corrected AIF (red). The same 20 table 1-aligned realizations were evaluated at every arterial oversaturation factor *f*; error bars are 95% confidence intervals. All returned parameter estimates were retained in the summaries.

Figure 5(B) evaluates the back-diffusion rate constant *k*_adb_. With the true AIF supplied to the fitter, mean ± SD absolute relative error ranged from 100 ± 200% at *f* = 2.0–100 ± 300% at *f* = 1.0 and was similarly large and variable with the corrected AIF. Thus, the available 40–55 s tissue curves did not reliably identify *k*_adb_, irrespective of AIF reconstruction. For *E*, corrected- and true-AIF fits were similar through *f* = 1.25, whereas an increasing downward shift appeared at higher *f*.

### 3.2. Human study

The correction framework was also applied to six unpaired human FVAs selected as illustrative examples: one undersaturated and one visibly oversaturated AIF from each of the control, DMnoDR, and mild NPDR groups. No independent reference AIF or ground-truth extraction fraction was available, so the cases were not treated as validation and no inferential test was performed. The arterial ROIs contained 175–655 pixels (median, 417 pixels; mean ± SD, 412 ± 162 pixels). The tissue ROIs contained 751–6176 pixels (median, 1843 pixels; mean ± SD, 2432 ± 1979 pixels). Correction produced little change in the three undersaturated examples. In the three oversaturated examples, the reconstructed AIFs and fitted *E* values changed, but the direction or magnitude of that change cannot establish improved accuracy (figures 6 and 7). The reconstructed Control 2 AIF extended beyond the empirically evaluated range of *f* ≤ 2.25; it is retained only as an exploratory out-of-range example and is not used to support the conclusions.

**Figure 6.**
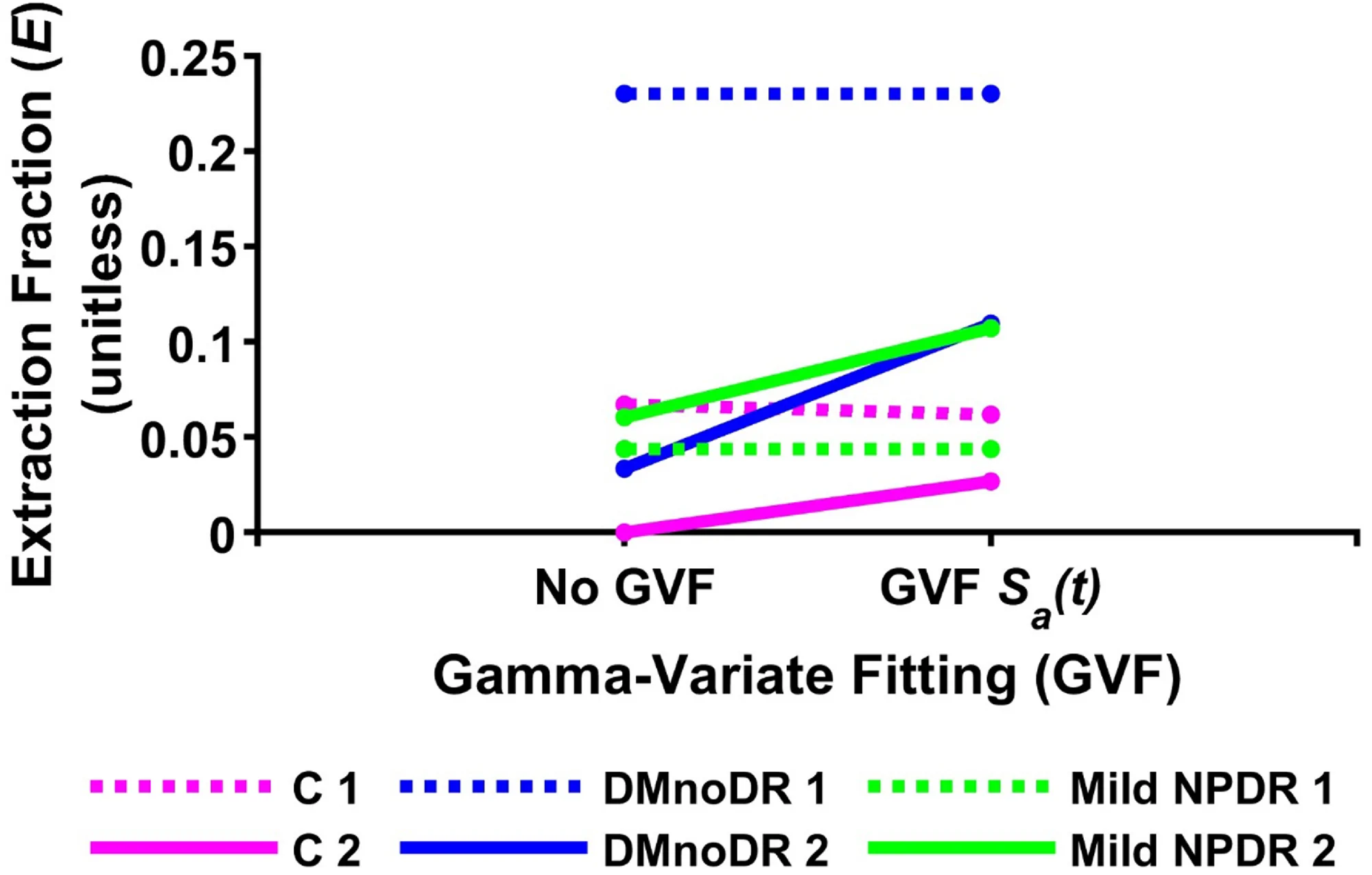
Extraction fraction (*E*) estimates from six illustrative human FVAs: one undersaturated (dashed line) and one visibly oversaturated (solid line) example from each diagnostic group: control (pink), DMnoDR (blue), and mild NPDR (green). Values are shown before and after arterial input function correction (i.e. no gamma-variate fitting (GVF) and GVF, respectively) to illustrate the within-case effect of the procedure. The oversaturated Control 2 example extends beyond the empirically evaluated range of *f* ⩽ 2.25 and is shown as an exploratory out-of-range case.

**Figure 7.**
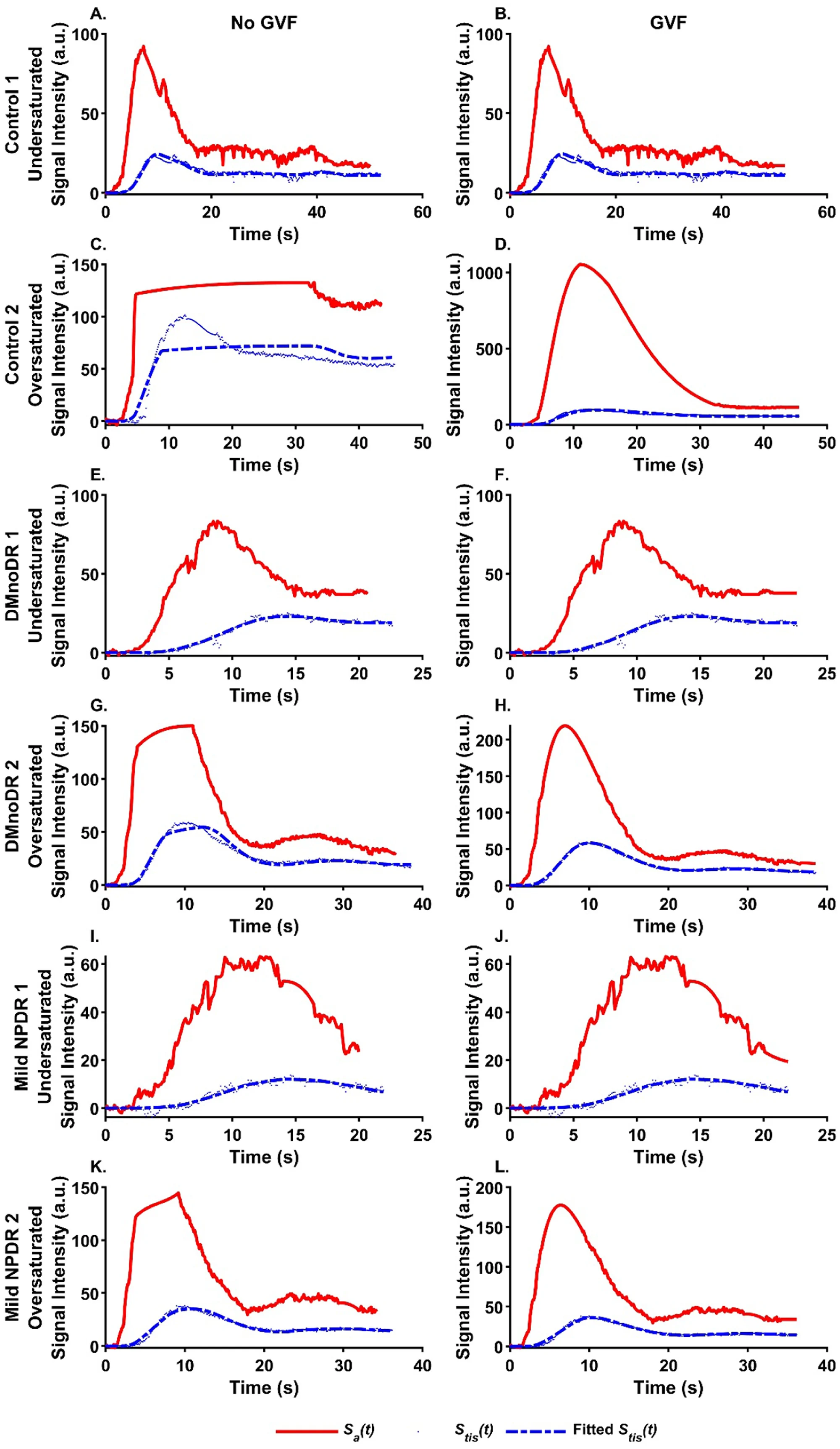
Representative tissue-curve fits for the six illustrative human FVAs shown in figure 6. Panels show one undersaturated and one visibly oversaturated example from each diagnostic group before and after AIF correction (i.e. no GVF and GVF, respectively), illustrating the resulting changes in the reconstructed AIF, tissue fit, and *E* estimate.

## 4. Discussion

This study evaluated a practical single-bolus framework for correcting first-pass AIF saturation in retinal FVA. In simulations, use of the raw saturated AIF produced increasing *E* error as saturation increased, whereas the combined truncated-mean and gamma-variate method retained lower error over modest oversaturation. Independent artificial clipping of measured AIFs supported the same reconstruction advantage without assuming synthetic pixel noise or spatial homogeneity. Under the tested simulation model, single-pixel fitting was unreliable at low signal but had no flagged failures once *f* reached 1. Overlapping confidence intervals did not identify a unique optimal *f*. The results therefore support operation near the nominal saturation threshold with explicit correction as a safeguard, while arguing against uncontrolled severe oversaturation.

These findings are relevant because retinal FVA presents an unusually constrained dynamic-range problem (Tichauer *et al* 2015a, 2015b, Vavrek *et al* 2023, 2025b). Quantitative tracer-kinetic analysis requires both an arterial input curve and a downstream tissue curve, yet the arterial and tissue fluorescein signals differ substantially in magnitude (Meier and Zierler 1954). Although attenuation of fluorescence by hemoglobin in large retinal vessels reduces the apparent disparity between arterial and tissue signals (Vavrek *et al* 2025a), it does not eliminate it. As a result, the limited dynamic range of standard 8-bit clinical FVA systems can become a fundamental constraint on quantitative analysis (Vavrek *et al* 2023), particularly when signal is increased to support smaller ROIs or pixel-level mapping. The present results indicate that if arterial saturation is ignored, this tradeoff leads directly to model bias. When explicit AIF correction was applied, signal at or modestly above the nominal limit improved the numerical reliability of simulated single-pixel fitting and maintained the reliability of ROI tissue curve fitting, whereas more substantial oversaturation increased sensitivity to the reconstructed AIF shape.

Preserving *E* estimation is clinically important because *E* is the permeability-related model output that has shown sensitivity to retinal vascular differences in our previous FVA studies (Vavrek *et al* 2025b). Across the modest saturation range, corrected-AIF *E* estimates remained substantially closer to the generating values than estimates obtained using the raw AIF, supporting the method’s intended application. *E* represents the fraction of fluorescein extracted from the vasculature during a single pass and, within the Renkin–Crone framework, is related to the permeability–surface-area product and blood flow (Renkin 1959, Crone 1963); it is not itself a direct measurement of permeability or permeability–surface-area product. The present results therefore establish the robustness of model-derived *E* to modest AIF saturation without implying a new diagnostic threshold.

Several strategies have been proposed in other imaging modalities to address first-pass AIF saturation, including dual-bolus, dual-sequence, reference-region, and blind-estimation approaches (Christian *et al* 2004, 2008, Gatehouse *et al* 2004, Groothuis *et al* 2010, Ishida *et al* 2011, Kershaw and Cheng 2011). These approaches are not readily transferred to retinal FVA because dual injections add procedural and fixation demands, while reference-region and blind methods require tissue, timing, or scaling assumptions that are difficult to satisfy in the retina. Kernel-based simultaneous estimation from an image-derived blood curve and multiple tissue curves is another promising alternative, although its applicability to retinal FVA remains to be established (Zhu *et al* 2024). Polynomial, spline, and gamma-variate interpolation have also been used to repopulate saturated AIF phases (Brunecker *et al* 2007, Wang and Cao 2012, Li *et al* 2023). In the present application, the combined truncated-mean and gamma-variate method was designed to use residual unsaturated arterial pixels before interpolating the saturation-dominated first-pass interval.

Differences in fluorescence-detection efficiency between arterial and tissue ROIs, expected to arise largely from hemoglobin absorption of the excitation and emission light of sodium fluorescein (Delori *et al* 1978, Simunovic *et al* 2023), were represented in the simulations through *ζ*, a tissue-to-artery optical detection-efficiency ratio estimated from first-order Beer–Lambert law assumptions (Vavrek *et al* 2025b). The model treated *ζ* as time-invariant and spatially uniform within each simulated realization and therefore represented the net artery-to-tissue detection-efficiency difference rather than concentration-dependent self-absorption, vessel-diameter-dependent attenuation, or spatially resolved hemoglobin and partial-volume effects. The kinetic fit consequently estimated *Fζ* as a combined signal-domain scale and could not identify physical blood flow and optical attenuation separately. The original synthetic arterial-pixel ensemble also imposed independent signal-dependent noise around a common arterial mean and did not reproduce all within-vessel spatial structure. We therefore complemented the simulations with artificial clipping of raw arterial-pixel time–activity curves from 10 undersaturated FVAs without adding synthetic noise. This empirical experiment retained the measured combination of vessel geometry, partial-volume effects, illumination nonuniformity, motion and registration residuals, spatially varying attenuation, and measurement noise, thereby testing reconstruction performance in the presence of real spatial and optical heterogeneity. It could not, however, isolate the contribution of hemoglobin absorption from these other effects.

Direct ground-truth measurement of a saturated retinal AIF in humans is not currently available. The framework was therefore evaluated using three complementary levels of evidence. Paired simulations provided known arterial and kinetic generating values with which to evaluate *E* recovery; artificial clipping of raw arterial-pixel curves tested AIF reconstruction while retaining measured spatial heterogeneity and noise; and six human cases demonstrated practical application of the procedure to undersaturated and visibly oversaturated acquisitions. In the raw-pixel experiment, the original unclipped arterial ROI mean served as an empirical reference rather than noise-free ground truth. The six additional human cases did not have independent reference AIFs or known *E* values and were therefore used to illustrate the within-case effect of correction rather than to establish accuracy. The Control 2 reconstruction exceeded the empirically evaluated range of *f* ⩽ 2.25 and was retained only as an exploratory example.

The method is intentionally designed to correct saturation occurring during the first-pass arterial peak, where a gamma-variate representation is physiologically appropriate (Davenport 1983, Amartey *et al* 2024). Later measured AIF samples were left unchanged. Several source AIFs contained a late secondary increase, whereas others ended earlier or continued to decline; these features were present in the original human curves and were not introduced by the correction. If saturation were to extend beyond the first pass or obscure later recirculation features, a different interpolation model would be required. The current evidence base also included four development AIF templates, 20 paired kinetic realizations, and extrapolation of the synthetic noise relationship above its fitted intensity range. The independent 10-FVA raw-pixel experiment reduces reliance on these simulation assumptions, while future studies across additional subjects, acquisition systems, and detector characteristics will further establish generalizability. Finally, *k*_adb_ was not identifiable over the available 40–55 s acquisitions even when the true AIF was supplied to the fitter. The present study therefore establishes *E*, rather than *k*_adb_, as the supported kinetic endpoint; longer acquisitions and regularized or parameter-reduced models may enable evaluation of additional parameters.

Additional limitations include the small development set of four AIF templates, the 20-realization paired kinetic simulation, and extrapolation of the synthetic square-root noise relation above the ⩽120 a.u. fitting range. The separate 10-FVA raw-pixel validation reduces, but does not eliminate, these generalizability concerns. The implementation also depends on correct detection of saturated pixels, *q*_thresh_, and fitting-window selection. Join diagnostics showed small value mismatch but nonzero slope mismatch because derivative continuity was not imposed. Finally, *k*_adb_ was not identifiable over the 40–55 s acquisitions even with the true AIF, so accurate recovery of *E* cannot be taken to establish identifiability of all AATH parameters. Future work should examine longer acquisitions, regularized or parameter-reduced fitting, alternative detector characteristics, and prospective pixel-level repeatability.

Taken together, these results support operating at or near the nominal AIF saturation threshold. For ROI analysis, explicit AIF correction maintained extraction fraction accuracy through modest oversaturation, reducing the need to preserve complete AIF linearity at the expense of tissue signal. For single-pixel analysis, increasing signal toward the detector ceiling improved fitting reliability, and low extraction fraction error was maintained through modest oversaturation. The absence of a unique optimal *f* is not a practical weakness, because variation in physiology, bolus delivery, optical attenuation, and acquisition conditions would make a single target difficult to achieve prospectively. The principal contribution is therefore a broader and more forgiving operating range for existing 8-bit FVA systems, with the potential to support spatial extraction fraction mapping and reveal retinal vascular heterogeneity obscured by large-ROI averaging (Abbasnejad *et al* 2023).

## 5. Conclusion

A practical single-bolus framework for correcting first-pass AIF saturation in retinal FVA was developed and evaluated. By combining truncated-mean reconstruction of partially saturated arterial measurements with gamma-variate interpolation when direct reconstruction became unreliable, the method preserved extraction fraction (*E*) estimation accuracy through modest AIF oversaturation, whereas uncorrected saturation produced progressively greater error. Independent artificial clipping of raw arterial-pixel time curves from 10 FVAs further demonstrated improved first-pass AIF recovery relative to no correction, PCHIP, and gamma-variate interpolation alone. These findings are particularly valuable for current clinical FVA systems with only 8-bit intensity resolution, where restricting acquisition intensity to ensure complete AIF linearity can compromise tissue signal and make pixel-level analysis difficult. In the simulations, single-pixel *E* estimation improved as signal approached the nominal AIF saturation limit and remained reliable through modest oversaturation when correction was applied. The proposed method therefore broadens the usable operating range of retinal FVA near the detector ceiling, reducing the need to avoid AIF saturation at all costs and providing a practical foundation for higher-resolution *E* mapping. Within this operating range, AIF saturation becomes a correctable acquisition condition rather than a reason to reduce tissue signal or discard a study.

## Data Availability

The de-identified data and MATLAB code supporting the findings of this study are available from the corresponding author upon reasonable request, subject to institutional approvals and participant-consent restrictions.

## Data availability statement

The data cannot be made publicly available upon publication because the cost of preparing, depositing and hosting the data would be prohibitive within the terms of this research project. The data that support the findings of this study are available upon reasonable request from the authors.
